# Identification of genomic regions and candidate genes for chicken meat ultimate pH by combined detection of selection signatures and QTL

**DOI:** 10.1186/s12864-018-4690-1

**Published:** 2018-04-25

**Authors:** Elisabeth Le Bihan-Duval, Christelle Hennequet-Antier, Cécile Berri, Stéphane A. Beauclercq, Marie Christine Bourin, Maryse Boulay, Olivier Demeure, Simon Boitard

**Affiliations:** 1 BOA, INRA, Université de Tours, 37380 Nouzilly, France; 2 Institut Technique de l’Aviculture (ITAVI), Centre INRA Val de Loire, F-37380 Nouzilly, France; 3 Syndicat des Sélectionneurs Avicoles et Aquacoles Français (SYSAAF), Centre INRA Val de Loire, Unité de Recherches Avicoles, F-37380 Nouzilly, France; 4 PEGASE, Agrocampus Ouest, INRA, 35590,, Saint-Gilles, France; 5 Groupe Grimaud, La Corbière, 49450, Roussay, France; 6 GenPhySE, Université de Toulouse, INRA, ENVT, 31320 Castanet Tolosan, France

**Keywords:** Chicken, Meat ultimate pH, Muscle glycogen, QTL, Selection signatures

## Abstract

**Background:**

The understanding of the biological determinism of meat ultimate pH, which is strongly related to muscle glycogen content, is a key point for the control of muscle integrity and meat quality in poultry. In the present study, we took advantage of a unique model of two broiler lines divergently selected for the ultimate pH of the pectoralis major muscle (PM-pHu) in order to decipher the genetic control of this trait. Two complementary approaches were used: detection of selection signatures generated during the first five generations and genome-wide association study for PM-pHu and Sartorius muscle pHu (SART-pHu) at the sixth generation of selection.

**Results:**

Sixty-three genomic regions showed significant signatures of positive selection. Out of the 10 most significant regions (detected by HapFLK or FLK method with a *p*-value below 1e-6), 4 were detected as soon as the first generation (G1) and were recovered at each of the four following ones (G2-G5). Another four corresponded to a later onset of selection as they were detected only at G5. In total, 33 SNPs, located in 24 QTL regions, were significantly associated with PM-pHu. For SART-pHu, we detected 18 SNPs located in 10 different regions. These results confirmed a polygenic determinism for these traits and highlighted two major QTL: one for PM-pHu on GGA1 (with a Bayes Factor (BF) of 300) and one for SART-pHu on GGA4 (with a BF of 257). Although selection signatures were enriched in QTL for PM-pHu, several QTL with strong effect haven’t yet responded to selection, suggesting that the divergence between lines might be further increased.

**Conclusions:**

A few regions of major interest with significant selection signatures and/or strong association with PM-pHu or SART-pHu were evidenced for the first time in chicken. Their gene content suggests several candidates associated with diseases of glycogen storage in humans. The impact of these candidate genes on meat quality and muscle integrity should be further investigated in chicken.

**Electronic supplementary material:**

The online version of this article (10.1186/s12864-018-4690-1) contains supplementary material, which is available to authorized users.

## Background

In chicken as in pigs, muscle ultimate pH (pHu) is a major factor of variation of both meat quality and processing ability. Normal values of pHu of broiler breast meat are approximately 5.8 to 5.9. The further the pHu deviates from this value, the more quality defects occur. Meat with pHu values greater than 6.1 is classified as dark, firm, and dry (DFD)-like meat while meat with pHu values less than 5.7 is classified as acid meat which is often referred to as pale, soft, and exudative (PSE)-like meat in broiler [1]. These quality defects lead to economic losses induced by reduced water holding capacity, increased cooking loss, decreased tenderness, and reduced emulsification capacity in the case of acid meat. In case of DFD-like meat, atypical color, after-flavor, dry and sticky texture, and decreased product shelf life are observed [2]. The near perfect genetic correlation (− 0.97) between pHu and glycogen content of breast muscle highlights a common genetic background between the two traits [3]. Glycogen is the major storage form for carbohydrates particularly in the liver and skeletal muscle [4]. In chicken, selection for increased growth rate and breast muscle mass has been associated with reduced glycogen storage [3, 5, 6]. Moreover, recent studies have reported reduced muscle glycogen content or elevated ultimate pH in breast muscles affected by degenerative disorders such as white striping and wooden breast [7, 8]. The understanding of the biological determinism of glycogen content and ultimate pH is thus a key point for the control of muscle integrity and meat quality in chicken.

It is now well established that chicken breast meat ultimate pH has a moderate to high heritability, as shown by the heritability values of 0.3 to 0.5 found in different genetic lines [3, 9–12]. However, in contrast to pigs where numerous QTLs (Animal QTLdb) and a major gene [13] have been identified for pHu, the genetic architecture of this trait remains poorly understood in chicken.

**Fig. 1:**
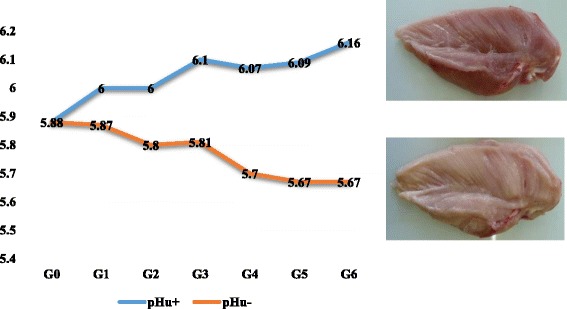
Phenotypic evolution of the ultimate pH of the pectoralis major (breast) muscle according to the generation of selection

In the present study, we took advantage of a unique resource population constituted from two lines divergently selected for pHu, to identify the genomic regions underlying the genetic variability of this trait and to search for candidate genes. As described in Alnahhas et al. [14], the two lines have been divergently selected since 2009 with the breeding value of the pHu of the pectoralis major muscle (PM-pHu) as selection criterion. This population originated from a grandparental fast-growing line of broiler chicken, selected for a balance between growth and reproduction traits. As the measure of PM-pHu requires sacrificing the birds, a sib-selection was applied. As shown in Fig. 1, the selection process has been quite efficient. After six generations of selection, mean PM-pHu was estimated at 5.67 in the pHu- line while it was equal to 6.16 in the pHu + line (*p*-value < 0.0001). This means that 61% of the breast meat in the pHu- line could be classified as acid or PSE-like (PM-pHu < 5.7) and 63% of breast meat in the pHu + line as DFD (PM-pHu > 6.1). Significant changes (*p*-value < 0.0001) were also observed in the thigh since the pHu of the Sartorius muscle was on average 6.20 and 6.53 in the pHu- and pHu + lines, respectively. As expected, by comparison to the PM muscle of the pHu + line, the PM muscle of the pHu– line was characterized by a higher Glycolytic potential [14]. In order to identify the genomic regions potentially affected by this divergent selection, a genome-wide scan for loci with outstanding genetic differentiation between the two lines was performed on the first five generations of selection. Although the divergent selection focused on a specific trait, several other traits diverged between the two lines. This was the case, as expected, for several parameters of meat quality (such as color, drip loss, texture) but also for the percentage of meat (thigh plus breast) which was higher in the pHu + line than in the pHu- line (14). Consequently, it was not possible to establish a direct relationship between particular selection signatures and the phenotypic expression of PM-pHu. Therefore, genome-wide QTL mapping was conducted on more than 550 birds from the sixth generation of selection to identify genomic regions with direct impact on the ultimate pH of the breast and the thigh meat, two traits which are known to be strongly genetically correlated [14].

## Results

### Selection signatures

We looked for genomic signatures of positive selection in the two lines using the FLK [15] and hapFLK [16] methods. These methods consider genotype data from multiple populations and detect regions where genetic differentiation between these populations is too large to result from a neutral evolution model. To do so, they proceed in three steps. First, they estimate the inbreeding coefficient in the different sampled populations, which quantifies the amount of drift that has been accumulated in these populations since their divergence from a common ancestral population. They do it based on genome-wide data, assuming that most loci genome-wide have evolved neutrally, so that the few loci that have evolved under selection have little influence on this estimation. Second, for each genotyped locus, they compute a *p*-value measuring how likely it is to reach the level of genetic differentiation observed at this locus, under a pure drift model with the inbreeding values estimated previously. Third, based on these *p*-values, they detect outlier loci using a standard statistical procedure controlling the false discovery rate (FDR). Using this approach, even strong levels of genetic differentiation may be considered non-significant, depending on the level of genetic drift estimated genome-wide. For the second step (the computation of *p*-values), genetic differentiation at each locus is evaluated based on single SNP allele frequencies for FLK, and on local haplotype frequencies for hapFLK. Haplotype data is expected to provide higher detection power, especially with medium density chip data [16]. More details about FLK and hapFLK can be found in the Methods.

In our analysis, the estimated inbreeding coefficient F from generation 0 to generation 5 was 0.08 in the pHu- line and 0.068 in the pHu + line. In a Wright-Fisher model with N diploïd individuals, F is equal to (1-(1–1/2 N)^g), where g is the number of generations. Based on this formula with g = 5, these estimations correspond to an effective population size of 30 animals in the pHu- line and 35 animals in the phu + line, which is in good agreement with the number of reproducers used at every generation of the experiment (see the Methods).

**Fig. 2:**
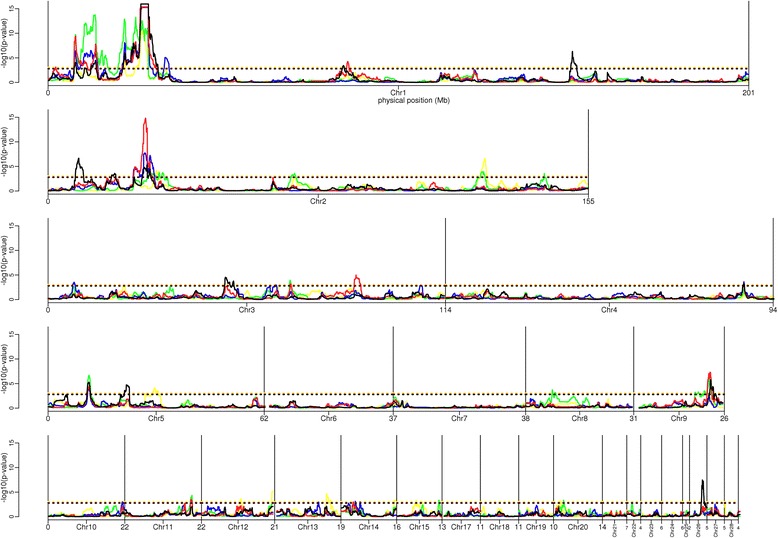
*p*-values (in -log10 scale) obtained along the genome when applying hapFLK to generation 1 (yellow), 2 (green), 3 (blue), 4 (red) or 5 (black) of the selection experiment. Horizontal dotted lines indicate the 5% FDR threshold for each of these generations. Vertical black lines correspond to a change of chromosome, and the numbers below these lines provide the length of the finishing chromosome

**Fig. 3:**
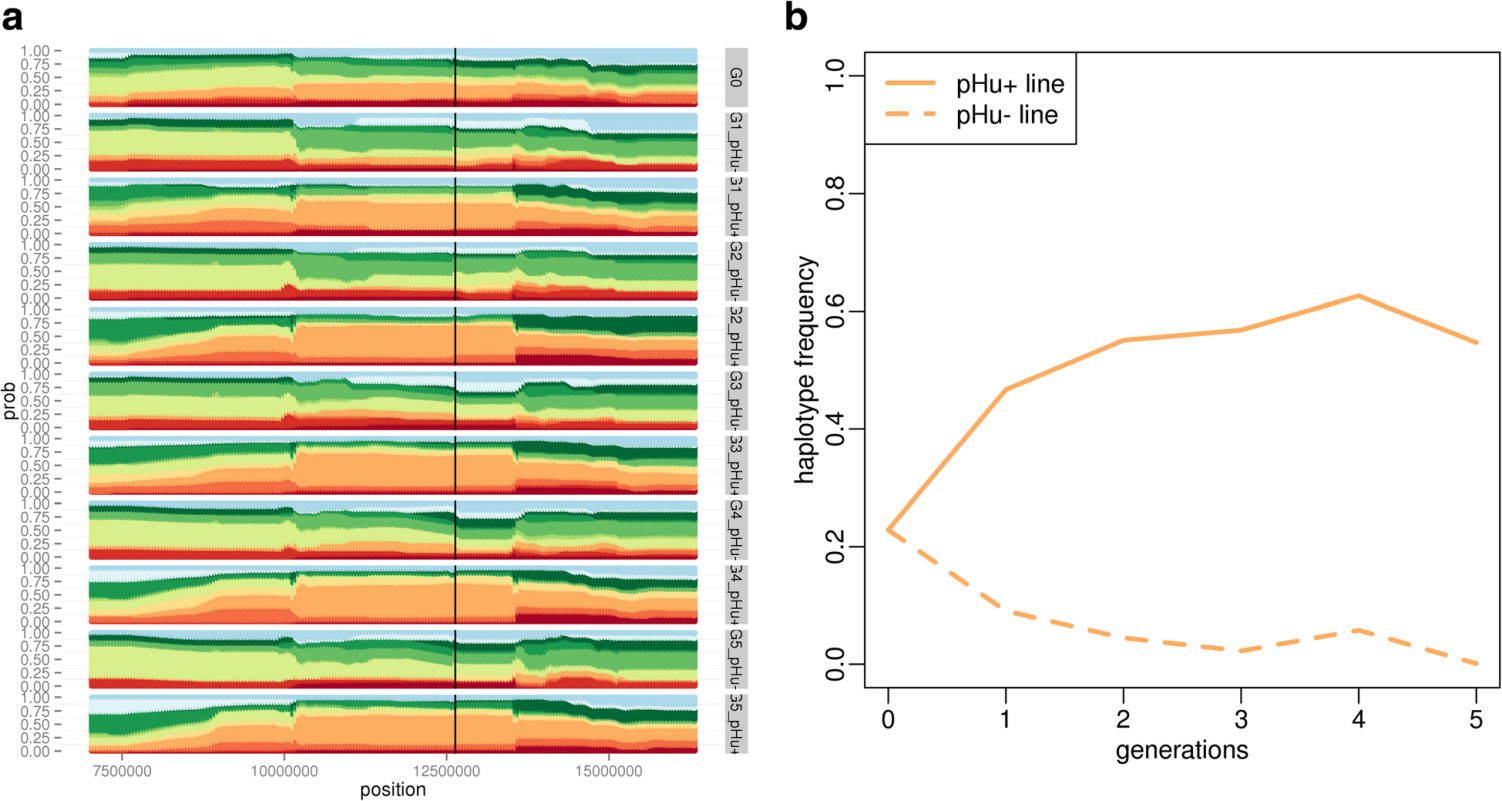
Evolution of haplotype cluster frequencies in region hapFLK-1b (Chromosome 1, from 7,050,235 to 16,305,108 bp). A: each panel corresponds to a population, from G0 (top) to G5_pHu + (bottom). For one given genomic position (on the x-axis), each color band corresponds to one haplotype cluster, and the height of this band gives the cluster frequency. The selection scenario described in the text is based on cluster frequencies at the position of the strongest hapFLK signal, which is indicated by the vertical black line. B: Evolution of the orange cluster frequency along generations. As discussed in the text, this cluster is the one showing the strongest evidence of selection at this locus

A total of 107 significant regions were detected with hapFLK at a FDR of 5%, when considering the different generations of the experiment (Fig. 2). Merging overlapping and contiguous signals found at different generations, we finally obtained 53 candidate regions (Table 1) under positive selection between generations 0 and 5. These regions were rather evenly distributed across the genome but their size varied from 30 kb to more than 11 Mb. Three regions (named hapFLK-1b, hapFLK-1c and hapFLK-2c in Table 1) were particularly outstanding, with *p*-values below 1e-14. Applying FLK to the different generations at a FDR of 5% (Additional file 1: Figure S1), we confirmed four of the regions already detected by hapFLK (hapFLK-1c, hapFLK-2a, hapFLK-3b, hapFLK-26a), and identified ten additional regions under selection (Table 2). In order to characterize the selection process in candidate regions, we plotted the evolution of haplotype frequencies between generation 0 (G0) and generation 5 (G5) in these regions. This revealed contrasted selection scenarios, as can be seen for the three strongest hapFLK signals. In region hapFLK-1b (Fig. 3), one haplotype (in orange) segregating at moderate frequency in G0 (about 25%) spread in the pHu + line, reaching a frequency of about 55% in G5, and was almost lost in the pHu- line. As a result, genetic diversity was greatly reduced in the pHu + line, while remaining quite high in the pHu- line. In region hapFLK-1c (Additional file 2: Figure S2), the contrast between the lines was even larger: one haplotype (in red) was almost fixed in the pHu- line and almost eliminated from the pHu + line, while a group of two other haplotypes (in green and dark red) increased to a frequency of 75% in the pHu + line and almost disappeared from the pHu- line. In region hapFLK-2c (Additional file 3: Figure S3), selection also led to the spread of one specific haplotype in each of the two lines (in light blue for pHu- and in light green for pHu+). Contrary to the situations described above, the two selected haplotypes were segregating at low frequency in G0 (7% for the blue one and 14% for the green one) and reached only intermediate frequencies in G5 (37% for the blue one and 28% for the green one). In the classical selection terminology, the selection events occurring in this region, especially the one concerning the light blue cluster, could be defined as incomplete hard sweeps, and the ones occurring in regions hapFLK-1b and hapFLK-1c as soft sweeps [17].

**Table 1:** Selection signatures detected by hapFLK across the five generations of selection

Name	Chr	Start^a^	End^a^	Length (Mb)	Marker-start	Marker-end	-log10 (pvalue)	generations
hapFLK-1a	1	2,276,648	2,445,683	0,17	Gga_rs13824804	GGaluGA002933	3.1	4
hapFLK-1b	1	7,050,235	16,305,108	9,25	Gga_rs13826175	Gga_rs14792165	13.8	1/2/3/4/5
hapFLK-1c	1	18,755,135	29,764,967	11,01	Gga_rs13834995	GGaluGA010403	16	1/2/3/4/5
hapFLK-1d	1	31,514,282	32,835,659	1,32	Gga_rs15226289	GGaluGA011179	5.1	3
hapFLK-1e	1	81,104,398	82,966,198	1,86	Gga_rs15322461	GGaluGA028823	4.2	4/5
hapFLK-1f	1	145,728,258	147,459,365	1,73	Gga_rs13953792	Gga_rs14902012	6.3	5
hapFLK-2a	2	7,923,417	10,281,724	2,36	Gga_rs15881724	Gga_rs14137940	6.7	5
hapFLK-2b	2	18,276,719	19,514,706	1,24	GGaluGA135806	Gga_rs14147239	3.6	5
hapFLK-2c	2	24,502,152	33,353,778	8,85	GGaluGA138005	GGaluGA140460	14.8	1/2/3/4/5
hapFLK-2d	2	68,724,706	70,032,818	1,31	Gga_rs16032833	Gga_rs14203112	3.6	2
hapFLK-2e	2	118,779,538	121,679,302	2,90	Gga_rs14241471	Gga_rs13730111	6.6	1/2
hapFLK-2f	2	136,816,316	137,093,235	0,28	Gga_rs15168561	GGaluGA170627	3.6	2
hapFLK-3a	3	7,225,785	7,845,634	0,62	Gga_rs14318013	GGaluGA205732	3.5	3
hapFLK-3b	3	48,835,040	51,520,696	2,69	Gga_rs14356552	GGaluGA222074	4.5	5
hapFLK-3c	3	62,672,584	63,014,334	0,34	Gga_rs14367181	Gga_rs14367507	3	3
hapFLK-3d	3	66,769,291	67,246,937	0,48	GGaluGA226759	GGaluGA226948	3.9	2/4
hapFLK-3e	3	85,356,860	87,242,706	1,89	Gga_rs16314104	Gga_rs15417891	5	4
hapFLK-3f	3	104,431,751	104,471,024	0,04	Gga_rs16336587	GGaluGA237591	2.9	3
hapFLK-4a	4	82,885,618	83,329,800	0,44	Gga_rs14498140	Gga_rs14498744	3.6	3/4/5
hapFLK-5a	5	10,152,843	11,215,390	1,06	Gga_rs14513950	Gga_rs14514889	6.7	1/2/3/4/5
hapFLK-5b	5	19,178,024	19,259,330	0,08	Gga_rs14520166	Gga_rs14520241	2.8	5
hapFLK-5c	5	20,133,297	21,384,953	1,25	Gga_rs14520800	GGaluGA278428	4.7	5
hapFLK-5d	5	27,596,949	28,539,932	0,94	Gga_rs14526966	Gga_rs16483831	4.1	1
hapFLK-5e	5	28,673,701	29,366,436	0,69	GGaluGA281566	Gga_rs14528861	3.6	1
hapFLK-8a	8	7,263,002	7,612,534	0,35	Gga_rs14638726	GGaluGA325359	3.8	2
hapFLK-8b	8	8,018,303	8,405,519	0,39	Gga_rs14639635	Gga_rs13663458	3.2	2
hapFLK-9a	9	16,456,190	16,641,999	0,19	GGaluGA341646	GGaluGA341716	3.2	2
hapFLK-9b	9	16,974,003	17,732,211	0,76	GGaluGA341780	GGaluGA341981	3.3	2
hapFLK-9c	9	18,382,877	18,479,085	0,10	Gga_rs16675119	Gga_rs14678141	2.9	2
hapFLK-9d	9	18,631,820	19,401,339	0,77	Gga_rs16675279	GGaluGA342447	3.5	2
hapFLK-9e	9	19,461,244	21,431,722	1,97	GGaluGA342478	Gga_rs16678760	7.3	2/4/5
hapFLK-9f	9	21,879,722	22,086,546	0,21	Gga_rs15985674	GGaluGA343636	3	5
hapFLK-10a	10	19,081,523	19,299,306	0,22	Gga_rs10723404	GGaluGA072999	3	3
hapFLK-11a	11	17,073,257	17,609,983	0,54	Gga_rs14027883	Gga_rs14028221	4.4	2/3/4
hapFLK-12a	12	10,796,360	11,156,696	0,36	Gga_rs14979504	Gga_rs14041284	3.7	1
hapFLK-12b	12	19,481,668	19,919,088	0,44	Gga_rs15675057	Gga_rs15675542	5.1	1/2
hapFLK-13a	13	12,453,968	12,578,242	0,12	Gga_rs14060968	Gga_rs15701870	3	3
hapFLK-13b	13	14,544,320	15,159,550	0,62	Gga_rs15704836	Gga_rs15705956	4.7	1
hapFLK-13c	13	15,240,280	15,290,260	0,05	Gga_rs15706156	GGaluGA097106	3.2	1
hapFLK-13d	13	15,616,564	15,746,776	0,13	GGaluGA097221	Gga_rs14063767	3.4	1
hapFLK-14a	14	3,483,024	3,608,186	0,13	Gga_rs14071682	Gga_rs14071828	3	4
hapFLK-14b	14	4,115,926	4,227,946	0,11	Gga_rs14072331	Gga_rs15725549	3.1	3
hapFLK-14c	14	5,463,733	5,536,467	0,07	Gga_rs14073705	Gga_rs15007686	3	2/3
hapFLK-14d	14	14,443,050	14,996,320	0,55	Gga_rs16702231	Gga_rs16015979	3.4	1
hapFLK-15a	15	5,559,590	5,671,833	0,11	GGaluGA108081	Gga_rs15020764	3.4	1
hapFLK-15b	15	7,076,061	7,108,783	0,03	Gga_rs14091544	Gga_rs13527470	3.1	1
hapFLK-15c	15	11,807,086	11,997,509	0,19	Gga_rs15786648	Gga_rs15787069	3.3	1
hapFLK-20a	20	1,850,026	2,053,990	0,20	GGaluGA175409	GGaluGA175568	3.3	1
hapFLK-20b	20	2,982,158	3,193,898	0,21	GGaluGA176153	Gga_rs16161873	3.4	2
hapFLK-22a	22	1,552,061	1,609,384	0,06	GGaluGA185952	Gga_rs16688631	3	2
hapFLK-26a	26	3,525,075	4,343,150	0,82	Gga_rs14299983	GGaluGA197644	7.5	5
hapFLK-26b	26	4,439,104	4,504,187	0,07	GGaluGA197707	Gga_rs15236117	3.1	5
hapFLK-26c	26	4,700,655	4,736,272	0,04	Gga_rs10724076	Gga_rs14301155	2.8	5

^a^ Positions are indicated on galgal5 assembly

**Table 2:** Additional selection signatures detected by FLK across the five generations of selection

Name	Chr	Start^a^	End	Length (Mb)	Marker-start	Marker-end	-log10 (*p*value)	generations	Nb. Significant SNP	BF GWAS^b^
FLK-2a	2	5,149,709	7,570,647	2.42	Gga_rs14131676	GGaluGA132502	4.2	5	1	3.7
FLK-2b	2	91,974,519	93,901,297	1.93	Gga_rs14219779	GGaluGA158657	4.8	5	12	1.2
FLK-3a	3	40,294,349	42,270,023	1.98	GGaluGA218034	Gga_rs13782352	4.6	5	1	3.2
FLK-8a	8	19,374,476	21,341,372	1.97	Gga_rs15922613	Gga_rs16638942	4.6	5	1	24.1
FLK-8b	8	27,320,551	29,297,449	1.98	Gga_rs14656403	GGaluGA333330	4.5	4/5	1	5.8
FLK-11a	11	11,744,741	14,765,393	3.02	GGaluGA077714	GGaluGA078384	4.7	5	3	9.6
FLK-13a	13	1,467,325	4,738,749	3.27	Gga_rs14049759	Gga_rs14054446	5.2	5	1	11.5
FLK-13b	13	9,083,535	12,423,603	3.34	Gga_rs16700430	GGaluGA095616	8.4	5	9	2.4
FLK-17a	17	6,849,442	9,763,118	2.91	Gga_rs14100058	Gga_rs15027305	4.8	5	2	10.8
FLK-24a	24	2,969,632	4,863,150	1.89	Gga_rs16195909	Gga_rs14297847	4.6	5	1	2

^a^Positions are indicated on galgal5 assembly

^b^Largest Bayes Factor for the association with the ultimate pH of pectoralis major (PM-pHu), for a model without line effect, among the SNPs in the region

### QTL of breast and thigh ultimate pH

Based on 558 animals sampled in the two lines at generation 6, we looked for QTL of breast and thigh ultimate pH, using the Bayes Cπ approach. Similar to the approaches used to detect selection signatures, Bayes Cπ is known to be robust to population structure effects (see the Methods). A total of 33 SNPs from 24 QTL regions were found significantly associated with the ultimate pH of the pectoralis major (breast) muscle in the whole population constituted from the two divergent lines (Table 3). Out of these 33 SNPs, 24 showed suggestive association with pHu (3 < BF ≤ 20) and 9 showed strong association with pHu (BF > 20), when including line as fixed effect, indicating that they also contributed to the within line variability of PM-pHu. This was the case for the very strong SNP (BF = 300 among the two lines) found on GGA1 (Additional file 4: Figure S4) and for other marked peaks detected on the genome, such as on GGA2 (BF = 107 among the two lines) or GGA24 (BF = 110 among the two lines). Alnahhas et al. [14] have shown a significant genetic correlation (0.54) between breast and thigh ultimate pH in both lines. In the same study, the between-lines difference of 0.5 units of pHu observed in breast muscle was associated with a between-lines difference of 0.3 units of pHu in thigh muscle after 6 generations of selection. In the present study, 18 SNPs, belonging to 10 different regions, were associated with the ultimate pH of the Sartorius (thigh) muscle (Table 3). A very strong SNP (BF = 257) was detected on GGA4 (Additional file 5: Figure S5), while SNPs located in other QTL regions remained at a lower level of significance (22 < BF < 50). As for PM-pHu, all these SNPs remained at least suggestive (3 < BF ≤ 20) and for 9 of them strong (BF > 20) or very strong (BF > 150) in the within-line analysis. A few co-localizations were evidenced between QTL of breast and thigh pHu, such as on GGA1 (QTL PM-1a and SART-1a) and on GGA21 (QTL SART-21a and PM-21a). However, most QTL were detected only for breast or for thigh, which suggested that the effect of some genes was specifically exerted on the metabolism of one muscle or the other.

**Table 3:** List of QTL detected for the ultimate pH of pectoralis major (PM-pHu) and sartorius (SART-pHu) muscles

Name	chr	marker	Position^a^	Trait	BF Statistics^b^	Corresponding selection signatures
PM-1a	1	GGaluGA008928	24,859,232	PM-pHu	25,8 (3,5)	hapFLK-1c
SART-1a	1	Gga_rs13840996	25,657,556	SART-pHu	22,6 (7,2)	hapFLK-1c
SART-1a	1	Gga_rs14801623	25,685,185	SART-pHu	39,8 (8,0)	hapFLK-1c
PM-1a	1	Gga_rs15214395	25,810,165	PM-pHu	47,6 (23,4)	hapFLK-1c
SART-1a	1	Gga_rs13841091	25,825,380	SART-pHu	26,6 (7,1)	hapFLK-1c
SART-1a	1	Gga_rs14801760	25,851,776	SART-pHu	25,1 (7,0)	hapFLK-1c
PM-1a	1	Gga_rs14802541	26,664,752	PM-pHu	36,6 (6,3)	hapFLK-1c
PM-1a	1	Gga_rs13842050	26,895,425	PM-pHu	300,2 (23,7)	hapFLK-1c
PM-1b	1	GGaluGA012178	35,189,134	PM-pHu	33,6 (5,0)	–
PM-1c	1	GGaluGA027506	80,195,898	PM-pHu	24,4 (33,7)	hapFLK-1e
SART-1b	1	Gga_rs13583913	82,528,289	SART-pHu	41,5 (15,5)	hapFLK-1e
SART-1c	1	GGaluGA031114	88,589,870	SART-pHu	22,3 (20,2)	–
SART-1c	1	Gga_rs14856430	88,742,249	SART-pHu	50,3 (24,4)	–
SART-1d	1	Gga_rs13920866	109,502,008	SART-pHu	30,2 (20,2)	–
PM-2a	2	Gga_rs14135370	8,052,991	PM-pHu	107,0 (27,7)	hapFLK-2a
PM-2a	2	Gga_rs14135839	8,512,020	PM-pHu	25,2 (18,4)	hapFLK-2a
PM-2a	2	GGaluGA132849	8,557,078	PM-pHu	44,9 (16,6)	hapFLK-2a
PM-2b	2	Gga_rs13709509	66,672,738	PM-pHu	20,4 (18,2)	–
PM-2c	2	Gga_rs15126247	88,786,601	PM-pHu	90,5 (25,8)	–
PM-2d	2	Gga_rs16102256	110,593,876	PM-pHu	65,0 (34,8)	–
SART-3a	3	Gga_rs14408993	105,711,393	SART-pHu	28,7 (12,6)	–
SART-4a	4	Gga_rs16349591	1,914,424	SART-pHu	257,2 (211,7)	–
SART-4a	4	GGaluGA241837	1,935,633	SART-pHu	134,7 (93,0)	–
SART-4a	4	Gga_rs14419109	1,985,419	SART-pHu	30,5 (36,7)	–
SART-4b	4	Gga_rs16407811	50,234,136	SART-pHu	23,4 (19,8)	–
SART-4b	4	Gga_rs14467373	50,260,314	SART-pHu	23,2 (19,3)	–
SART-4c	4	Gga_rs14480120	62,306,853	SART-pHu	24,1 (14,7)	–
PM-4a	4	GGaluGA270819	90,110,928	PM-pHu	25,8 (5,8)	–
PM-5a	5	GGaluGA274824	10,559,934	PM-pHu	21,2 (4,4)	hapFLK-5a
PM-6a	6	Gga_rs13567905	16,876,982	PM-pHu	25,1 (9,9)	–
PM-6b	6	Gga_rs16552220	20,899,730	PM-pHu	24,5 (6,0)	–
PM-7a	7	GGaluGA308703	2,828,621	PM-pHu	34,6 (12,2)	–
PM-7b	7	Gga_rs15860854	22,215,517	PM-pHu	22,0 (3,4)	–
PM-8a	8	Gga_rs16631815	15,186,655	PM-pHu	21,5 (4,6)	–
PM-8b	8	Gga_rs13681628	20,157,176	PM-pHu	24,1 (8,3)	FLK-8a
PM-10a	10	Gga_rs14941507	967,702	PM-pHu	58,3 (18,0)	–
PM-10a	10	Gga_rs14001087	986,341	PM-pHu	20,5 (8,8)	–
PM-12a	12	Gga_rs14031079	1,528,565	PM-pHu	22,0 (8,0)	–
PM-12b	12	GGaluGA083170	5,811,993	PM-pHu	67,4 (7,6)	–
PM-12b	12	Gga_rs14035691	5,820,013	PM-pHu	88,1 (14,4)	–
PM-12c	12	Gga_rs14049172	19,145,586	PM-pHu	26,5 (24,8)	hapFLK-12b
PM-14a	14	Gga_rs14071447	3,274,074	PM-pHu	31,3 (8,2)	hapFLK-14a
PM-14a	14	GGaluGA100179	3,387,115	PM-pHu	80,9 (16,6)	hapFLK-14a
PM-16a	16	Gga_rs15026791	85,688	PM-pHu	34,6 (5,6)	–
SART-20a	20	Gga_rs13633029	6,881,983	SART-pHu	41,2 (34,3)	–
SART-20a	20	Gga_rs16167169	6,912,028	SART-pHu	35,6 (30,5)	–
PM-20a	20	Gga_rs15174825	7,499,329	PM-pHu	24,0 (6,1)	–
SART-21a	21	GGaluGA185204	5,971,877	SART-pHu	23,1 (21,6)	–
PM-21a	21	Gga_rs14285841	6,045,175	PM-pHu	34,3 (14,4)	–
PM-24a	24	Gga_rs13725186	6,092,691	PM-pHu	28,3 (24,4)	–
PM-24a	24	Gga_rs16199693	6,153,126	PM-pHu	110,5 (24,4)	–

^a^Positions are indicated on galgal5 assembly

^b^BF statistics obtained with a line effect fitted in the model are put in brackets

## Discussion

Chicken is a good model for studying genomic regions under selection. This is mainly due to the availability of valuable animal populations, such as divergent lines, and increasing genomic resources. The effects of single trait divergent selection have already been studied in this species with the 60 K SNP array. Johansson et al. [18] analyzed the genome of two divergent lines after 50 generations of selection for body weight and concluded that from 50 up to over 100 regions were under selection. Zhang et al. [19] considered a shorter term experiment, i.e. they looked for selection signatures after 11 generations of divergent selection for the percentage of abdominal fat and identified more than 50 regions within each of the two lines. In our study, even fewer generations (5) of divergent selection were considered, which should limit the detection of false positive selection signals due to genetic drift. We also controlled the risk of false positives by using statistical tests that explicitly estimate the amount of drift in sampled populations, and detect regions where genetic diversity is significantly inconsistent with a neutral model including this amount of drift. This resulted in the detection of 63 genomic regions under selection, at a false discovery rate (FDR) level of 5%. The inclusion of reference genotypes from G0 increased the detection power of this analysis. Indeed, the key point in hapFLK (resp. FLK) is to quantify, for each locus, the haplotype (resp. allele) frequency variation from an ancestral founder population to each sampled population (see the methods for more details). In most studies this ancestral population is unobserved, so ancestral frequencies are estimated from the frequencies in observed populations, but having direct access to these ancestral frequencies necessarily improves the approach.

Another original aspect of our study was to consider genotypes at successive generations, which allowed characterizing the dynamics of selection in regions underlying the genetic progress. Out of the 10 most significant hapFLK and FLK signals, with a *p*-value below 1e-6, four (named hapFLK-1b, hapFLK-1c, hapFLK-2c, hapFLK-5a in Table 1) were detected as soon as the first generation (G1) and were recovered at each of the four following generations (G2 to G5), and another one (hapFLK-9e) was detected at three generations (G2, G4 and G5). Four others (hapFLK-1f, hapFLK-2a, hapFLK-26a in Table 1 and FLK-13b in Table 2) corresponded to a later onset of selection, as they were detected only at G5. As shown in Table 1, various profiles were observed for the less significant selection signals, most of them being detected at only one generation and some others at 2 or 3 generations of the selection process. The dynamics of selection in candidate regions could also be visualized by plotting haplotype frequencies along generations in the two lines (Fig. 3, Additional file 2: Figure S2 and Additional file 3: Figure S3).

From a methodological perspective, our study also highlighted the interest of using haplotype instead of single SNP information for detecting selection signatures. Indeed, 53 candidate regions under selection were detected with hapFLK, with *p*-values as small as 1e-16 (Table 1), while only 14 were detected with FLK, with *p*-values down to 1e-8. The increased power provided by haplotypes has been demonstrated by Fariello et al. [16] using computer simulations, and has been confirmed since then by the outcomes of several applied studies in sheep [20], chicken [21] or cattle [22]. In their computer simulations, Fariello et al. [16] considered selection at a single SNP; in this case the higher power of hapFLK was related to the fact that the causal SNP itself was generally not genotyped, so that allele frequencies at this SNP (and their variation between populations) was better quantified using local haplotypes than other individual SNPs. However, as noted by the authors, another strong advantage of hapFLK is that selection can also act directly on a combination of linked alleles, due to epistatic effects; this situation is clearly easier to capture using haplotypes, because allele frequencies at each of the linked variants may only show minor variations.

Nevertheless, among the 14 regions detected by FLK, 10 were not detected by hapFLK (Table 2). This is a bit unexpected, because at the short time scale of our experiment recombination is limited, so the increase in frequency of one allele should be associated to that of a larger haplotype. In order to check that the FLK signal in these regions was not spurious, we considered the most significant SNP in each of the ten regions, and evaluated how likely it is that the allele frequency trajectories observed at this locus in the two lines, are reached due to drift. For this purpose, we used forward simulations without selection, but with population sizes mimicking our experiment (see the Methods for more details). When simulations were performed using a standard Wright-Fisher model, *p*-values remained small but generally increased, and some of them were no more significant (Additional file 6: Table S1). While the FLK and simulation approaches are both based on the allele frequency variation from generation 0 to 5, FLK *p*-values are computed by assuming that this variation follows a normal distribution. The accuracy of this approximation is known to decrease when the allele frequency in the ancestral population is close to 0 or 1, which is generally the case for the 10 SNPs considered here. This likely explains the difference between the two approaches. However, when considering a more realistic simulation model accounting for the exact number of male and female reproducers at each generation of the experiment, most *p*-values were found again very small. Besides, it should be noted that 7 of the 10 regions of Table 2 include several significant FLK SNPs or / and suggestive GWAS hits (BF > =5). Overall, we therefore believe that most of these 10 regions are true selective signals. The fact that they were not detected with hapFLK might be related to the difficulty of reconstructing haplotypes in regions where linkage disequilibrium is weak. Indeed, the average r2 within G0, for markers distant by less than 500 kb, was 0.442 (sd 0.005) for the 10 regions of Table 2, versus 0.467 (sd 0.0008) genome-wide and 0.512 (sd 0.004) for the 53 regions of Table 1. This suggests that combining hapFLK with FLK might still be useful in some cases, such as low SNP density or high recombination rate.

As a result of the divergent selection for PM-pHu, genomic regions showing selection signatures were enriched in QTL controlling this trait. On average, the Bayes Factor resulting from our association study in the whole population constituted from the two divergent lines was significantly larger for SNPs located within the candidate regions under selection of Tables 1 and 2, than for other SNPs in the genome (*p*-value of 0.0018 for the regions of Table 1 and 0.0078 for the regions of Table 2, with a two-sided Student test). Consistent figures were also found for the BF values issued from within-line analysis (*p*-value of 2.453e-06 for the regions of Table 1 and 0.04187 for the regions of Table 2). However, among the 24 QTL regions for PM-pHu trait of Table 3 (with BF > 20), only 7 corresponded to a selection signature (hapFLK-1c, hapFLK-1e, hapFLK-2a, hapFLK-5a, hapFLK-12b, hapFLK-14a, and FLK-8a). This is likely related to the polygenic architecture of PM-pHu, implying that QTL regions for this trait cannot all be efficiently selected in only 5 generations (at least not enough to be detected by our approach). Conversely, among the 63 regions under selection listed in Tables 1 and 2, only 7 corresponded to one of the 24 QTL regions for PM-pHu, and 8 others (including regions hapFLK-1b and hapFLK-2c discussed above) showed substantial evidence of association with PM-pHu (BF ≥ 5, data not shown). Thus, 48 regions with little evidence for association with PM-pHu showed significant selection signatures, and only 5% of those are expected to be false positives (see the methods). These regions likely correspond to QTL with relatively small effects, leading to moderate selection signatures. Indeed, as discussed previously, the use of individuals from G0 and, in the case of hapFLK, of haplotype information, allows detecting selection events with high power. In contrast, the power of the GWAS study is largely driven by single SNP allele frequency differences between the two lines, and moderate differences should not allow detecting association with PM-pHu. Finally, we cannot exclude the fact that traits or functions have been modified by the selection process although they are not directly related to muscle energy metabolism and meat ultimate pH.

The study of the two divergent lines allowed a better understanding of the genetic architecture of chicken meat ultimate pH and revealed several outstanding regions with highly significant signatures of selection and/or direct association with PM-pHu and SART-pHu phenotypes. In order to identify putative candidate genes, these regions were examined in light of the results of the transcriptomic analysis performed on samples from the pectoralis major muscle of the pHu + and pHu- lines [23] and of the knowledge of genes involved in human glycogen storage diseases (GSD) [24]. The most significant PM-pHu QTL identified in the current study (PM-1a) was located on GGA1 (BF = 300). It co-localized with a SART-QTL (named SART-1a) and with the most significant signature of selection detected by hapFLK at all generations of selection (named hapFLK-1c). The SNP exhibiting the strongest relation with PM-pHu (i.e. Gga_rs13842050) was segregating between the two lines, 86% of the pHu- birds being homozygous AA and 77% of the pHu + birds homozygous GG at the 6th generation of selection. It is likely that this SNP is in linkage disequilibrium with one or several mutations that affect PM-pHu. As shown in Additional file 7: Table S2, several differentially expressed (DE) genes were evidenced in this region. *PPP1R3A*, located ~ 506 kb away from the most significant SNP, is of special interest as it codes for a muscle-specific regulatory subunit of protein phosphatase 1 (PP1). By steering the catalytic subunit (PP1c) to glycogen, it promotes dephosphorylation of glycogen synthase (GS) and glycogen phosphorylase (GP) and thereby glycogen synthesis [24]. Knockout mice lacking *PPP1R3A* exhibit a 90% reduction in muscle glycogen [25]. A few years ago, a mutation was identified in human and was shown to be the first prevalent mutation known to impair glycogen synthesis and to decrease glycogen levels in human skeletal muscle [26]. *PPP1R3A* was significantly over-expressed in the pHu- line [23] which is consistent with the higher muscle glycogen content observed in this line [14]. Another gene, *SLC37A4,* involved in human GSD was present in the top list of the regions of interest (Additional file 7: Table S2). It is located ~ 507 kb away from one of the most significant SNP detected for PM-pHu on GGA24 (BF = 110). It corresponds to glucose-6-phosphate translocase (also called G6PT) which transports the glucose 6-phosphate from the cytoplasm to the endoplasmic reticulum, where it works together with the glucose 6-phosphatase to break down the sugar molecule and release free glucose that can leave the cell. Mutations in *SLC37A4* are estimated to account for about 20% of the GSD type I (or Von Gierke disease) characterized by the accumulation of glycogen in some organs and tissues [27]. As indicated in Additional 7 Table S2, several other DE genes were identified in the regions of interest. Two of them (*RHOC* and *LOC107052650*) were part of a set of 20 biomarkers evidenced as pertinent predictors of the variation of PM-pHu between the two lines [23]. This set also comprised *VTI1B*, *SLC2A1* and *CAV3* which were within signatures of selection or QTL regions detected at a lower level of significance in the current study (hapFLK-5e for *VTI1B*, QTL PM-21a for *SLC2A1*, and QTL PM-12c for *CAV3*). *SLC2A1*, also called *GLUT1*, is coding for Glucose transporter-1, which is considered to play a key role in maintaining basal glucose transport in most chicken cell types as in mammals [28]. Further investigation by eQTL detection should be envisaged for the most promising of these positional and expressional candidate genes. It is also worthwhile to note that two other genes responsible for human GSD [24] were present in the regions of interest even if they were not differentially expressed. This is the case for *GAA* (located within the QTL PM-14a) coding for lysosomal alpha-glucosidase which is essential for the degradation of glycogen to glucose in lysosomes and responsible for GSD type II or Pompe disease. It is also the case for *PHKA1* located ~ 20 kb away from the most significant SNP (BF = 257) we identified for SART-pHu on GGA4 (QTL SART-4a). The *PHKA1* gene encodes the muscle alpha regulatory subunit of phosphorylase kinase which catalyzes phosphorylation of glycogen phosphorylase and thus promotes glycogen degradation. Mutations in *PHKA1* cause GSD type VIII which is usually a mild myopathy with slight elevation of plasma creatine kinase concentration and muscle glycogen content.

## Conclusions

The divergent selection conducted on PM-pHu allowed the creation of a unique resource population. This population, as shown in the present study, is highly useful to understand the genetic control of not only the selection criterion but more generally that of muscle glycogen storage and metabolism in chicken. Sixty-three genomic regions showed significant signatures of positive selection and were enriched in QTL for PM-pHu. Still, several QTL with strong effect have not yet responded to selection, suggesting that the divergence between lines might be further increased. A few regions of major interest, with significant selection signatures all along the selection process and/or highly associated with PM-pHu or SART-pHu phenotypes, were evidenced for the first time in chicken. They suggested several candidate genes, directly involved in glycogen synthesis and degradation or in the balance between these two systems, which necessitate further investigation in chicken.

## Methods

### Birds and housing

This study was conducted on birds originating from two lines divergently selected for PM-pHu according to a breeding scheme described in Alnahhas et al. [14]. In this experiment, sires were selected with higher intensity: at each generation, the best 19% sires (i.e. with the highest genetic value for the selected trait) and 50% dams were chosen to produce the next generation. Consistent with this higher selection intensity, genotyping in the first 5 generations was focused on sires, as described below. At generation 0 (G0), where the divergence process was not yet started and all birds were reared as a single population, 51 sires were genotyped. At generations 1 to 5 (G1-G5), the number of sires with descendants varied between 26 and 29 in the pHu- line and between 23 and 31 in the pHu + line, and the number of these sires that were genotyped varied between 17 and 28 in the pHu- line and between 14 and 30 in the pHu + line. QTL detection by association study was performed on a total of 558 offsprings (253 males and 305 females) of the 6th generation of selection (G6). Birds were reared in two successive batches and phenotyped for PM-pHu and SART-pHu as described in Alnahhas et al. [29].

### Genotyping

Birds used for QTL mapping (*n* = 558) and sires used for the scan of positive selection (*n* = 288) were genotyped by the Labogena Laboratory (Jouy en Josas, France) using the Illumina chicken SNP 60 K Beadchip containing 57,636 SNPs. After filtering SNPs for their minor allele frequency (higher than 0.05) and their call rate (higher than 0.95), 40,590 SNPs located on 28 autosomes were retained for QTL detection on a total of 558 birds which had all a call rate higher than 0.95. When applying the same criteria, a total of 42,026 SNPs and 288 birds were considered for the detection of regions under selection.

### Statistical analyses

#### Detection of selection signatures

We looked for genomic signatures of positive selection using the FLK [15] and hapFLK [16] methods, both implemented in the hapFLK software (https://forge-dga.jouy.inra.fr/projects/hapflk). These methods consider genotype data from multiple populations and detect regions where genetic differentiation between these populations is not consistent with a neutral evolution model. First, based on the matrix of observed genome-wide pairwise Reynold’s distances between populations, they estimate a population tree, using a Neighbour-Joining algorithm, and summarize this tree by a kinship matrix quantifying the amount of drift accumulated in each population since their divergence from a common ancestral population. Then, they scan the genome for loci where genetic differentiation between populations is significantly larger than expected under this pure drift model. Similar to the classical Fst, the statistic measuring genetic differentiation for one allele is a sum of the terms (pi – p)^2, where pi is the frequency of this allele in population i and p the estimated frequency of this allele in the ancestral population. The difference with Fst is that the terms of this sum (i.e. the populations) are weighted using the entries of the kinship matrix estimated previously, in order to account for differences of population size and for shared ancestry between populations. In the case of FLK, the statistic is computed for one of the two alleles of each SNP (the choice is arbitrary and has no influence on the results). In the case of hapFLK, local haplotypes are estimated (see below) and genetic differentiation is summed over all these haplotypes.

We applied these two tests for all generations of the selection experiment. At each generation (G1 to G5), 3 populations were compared: the pHu + line, the pHu- line, and the G0 population. G0 was defined as outgroup when building the population tree and computing the kinship matrix (option –outgroup). Data from G0 were also used when computing genetic differentiation at each locus (option –keep-outgroup). As a result, estimated allele frequencies in the ancestral population were always very close to the allele frequencies observed in G0 (p0), so the allele frequency differences computed within FLK (the terms (pi-p)^2 mentioned above) were almost equal to the allele frequency differences (pi-p0)^2 observed along each selected line.

HapFLK makes use of the local clustering approach of Scheet and Stephens [30] to estimate haplotype diversity. This approach assumes that for each position on the genome, local haplotypes can be divided into K main clusters, where each cluster may include several similar haplotypes. As a result of ancestral recombinations, the pair of clusters corresponding to a given diploïd individual is allowed to change from one locus to the other, and is assumed to form a Markov chain along the genome. Using a hidden Markov model where these clusters are the hidden variables and individual genotypes are the observed variables, haplotype clusters at all loci can be estimated for all individuals. One advantage of this approach is that it avoids defining fixed windows where haplotypes should be estimated: instead, transitions of the Markov chain representing the local haplotype clusters of an individual are automatically learnt from the data. However, this approach requires specifying the number K of haplotype clusters. Applying the cross-validation procedure implemented in the fastPHASE software to genotype data from G0, we set this number to 12 (option -K). We inferred haplotype clusters using 50 Expectation-Maximisation (EM) runs (option –nfit). Following Fariello et al. [16, 20], we assumed that hapFLK statistic was normally distributed under neutral evolution, and estimated the parameters of this normal distribution by fitting genome wide hapFLK values with a robust linear regression approach. HapFLK *p*-values at each SNP were computed from this estimated distribution. To identify selected regions, we used a q-value threshold of 0.05, therefore controlling false discovery rate at the 5% level [31].

For FLK analyses, *p*-values were directly obtained from the hapFLK software (they can easily be computed because the distribution of the FLK statistics under neutral evolution was shown to follow a chi-squared distribution with known degrees of freedom). As described above, we used an FDR threshold of 5% to identify significant SNPs. In contrast to hapFLK values, which are relatively smooth along the genome, FLK values, as most single SNP statistics, are highly variable along the genome. Consequently, defining significant regions is more difficult in this case. However, in our analysis, only few SNPs had significant FLK values (1 in G4 and 57 in G5) and several of them were located in regions already detected by hapFLK. Based on significant FLK values that did not overlap with a hapFLK region, we defined 10 new regions under selection including either one significant SNP or several significant SNPs distant by less than 1 Mb.

For all regions detected under selection using FLK or hapFLK, we performed another hapFLK analysis combining animals from all generations, which allowed studying the evolution of haplotype cluster frequencies along generations (haplotype clusters obtained by independent hapFLK analyses are not comparable). For each region, we estimated haplotype clusters using 3 EM runs, and plotted cluster frequencies for each of these runs using the R script hapflk-clusterplot.R, which is provided on hapFLK website. These 3 runs always led to very similar interpretations concerning the selection history in the region, so we only presented one of them.

#### Forward simulations

In order to evaluate the likelihood of an allele frequency trajectory going from p0 to pf in 5 generations, we simulated 10 millions of 5 generation allele frequency trajectories starting from p0, recorded the final allele frequency pj in each simulation j, and computed the proportion of simulations such that (pj-p0)^2 was greater than (pf-p0)^2. Two simulation strategies were implemented. In the first strategy, we considered a standard Wright-Fisher model with N haploïd individuals: if one allele has x copies out of N individuals at generation g, the number of copies at generation (g + 1) is a binomial variable with parameters (N, x/N). In the second strategy, we accounted for the number of reproducing males Nm(g) and females Nf (g) at each generation g: if one allele has xm copies among the 2*Nm(g) male alleles and xf copies among the 2*Nf(g) female alleles at generation g, the number of copies among the males at generation (g + 1) is Xmm + Xmf, where Xmm is a binomial variable with parameters (Nm(g + 1), xm/2*Nm(g)) and Xmf is a binomial variable with parameters (Nm(g + 1), xf/2*Nf(g)). Similarly, the number of copies among the females at generation (g + 1) is Xfm + Xff, where Xfm is a binomial variable with parameters (Nf(g + 1), xm/2*Nm(g)) and Xff is a binomial variable with parameters (Nf(g + 1), xf/2*Nf(g)). For each simulation strategy, a first set of simulations was performed using parameters mimicking the pHu- line, and a second set was performed using parameters mimicking the pHu + line. For strategy 1, the number of haploïd individuals was the haploïd effective population size estimated by FLK. For strategy 2, the number of male and female reproducers at every generation was the same as in the divergent selection experiment. The final *p*-value at each SNP (provided in Additional file 6: Table S1) was the product of the likelihoods obtained in the two lines.

#### QTL detection

QTL detection on birds from generation 6 was performed by the multi-marker Bayes Cπ analysis [32] implemented in GS3 software [33]. The statistical model was:

$$ {y}_j=\mu +\sum \limits_{i=1}^M{x}_{ij}{a}_i+{e}_j $$

where *y*_*j*_ is the phenotype for an individual *j* corrected for the fixed effects of sex and hatch*, μ* the overall mean, *M* the number of markers analysed, *x*_*ij*_ the genotype score (coded as 0, 1 or 2) of SNP i for individual j, a_i_ the additive effect of SNP i, and *e*_*j*_ the random residual for individual j with ej ∼ N(0, **I**σ^2^_e_). **I** is an identity matrix and σ^2^_e_ is the residual variance. All unknown parameters were assigned prior distributions and sampled with a Monte Carlo Markov chain (MCMC) using Gibbs sampling. The MCMC was run for 400,000 iterations, with a burn-in of 80,000 iterations and thin interval of 400. The Bayes Cπ analysis allows introducing in the model all the SNPs at the same time but makes the assumption that only a small proportion of them has a significant effect on the trait. Thus, the prior parameter used for a_i_ is a mixture distribution with a_i_ ∼ N (0, σ^2^_a_) if the SNP is in the model (with a probability π) and a_i_ = 0 if the SNP is not in the model (with a probability 1-π). σ^2^_a_ is the common marker effect variance and the hyper parameter (1-π) the prior probability that the effect of marker *i* is 0. For π the prior distribution was set at a Beta distribution with parameters α = 0.5 × 10^4^ and β = 99.5 × 10^4^, meaning that π was almost fixed at 0.005. Variances σ^2^_a_ and σ^2^_e_ were assigned inverted chi-squared distributions with *v* = 4.2 degrees of freedom and a scale parameter ${S}^2=\frac{{\widehat{\sigma}}^2\left(\nu -2\right)}{\nu }$ where ${\widehat{\sigma}}^2$equals the prior value of σ^2^_a_ or σ^2^_e_. The statistics used to detect significant SNP was the Bayes Factor (BF), which corresponds to the increase from prior to posterior probabilities of the SNP being “in” the model [34]. In this study we retained markers corresponding to BF = 20–150 and BF > 150 which indicated strong and very strong evidence of QTL linkage, respectively [34]. We defined QTL regions by merging candidate SNPs distant by less than 1 Mb, and further extended each obtained region by including the two 1 Mb flanking regions (that is 1 Mb before the first SNP and 1 Mb after the last SNP).

By including all the SNPs simultaneously in the model while assuming that only a proportion of those (π) has a significant effect, Bayes Cπ ensures that strong BF can only be reached at SNPs where the association between genotypes and traits clearly exceeds that expected from population structure and cannot result from drift alone. BF statistics were also calculated with a model including the fixed effect of the line to test whether the SNPs contributing to the variability of PM-pHu or SART-pHu in the whole population constituted from the two divergent lines also contributed to the intra-line variability.

*Computation of linkage disequilibrium*

The linkage disequilibrium measure r2 was computed for all SNP pairs distant by less than 500 kb and with minor allele frequency greater than 0.1, using the –r2 command of the Plink software, version v1.90b3.34 (https://www.cog-genomics.org/plink2). This computation was performed using genotyped animals from generation 0. For a given category of regions (for instance those listed in Table 1), all SNP pairs belonging to this category were collected and the average r2 among these pairs was estimated.

## Additional files:

Additional file 1: **Figure S1.** *p*-values (in -log10 scale) obtained along the genome when applying FLK to generation 1 (yellow), 2 (green), 3 (blue), 4 (red) or 5 (black) of the selection experiment. Horizontal dotted lines indicate the 5% FDR threshold for each of these generations. Vertical black lines correspond to a change of chromosome, and the numbers below these lines provide the length of the finishing chromosome. (TIFF 1599 kb)

Additional file 2: **Figure S2.** Evolution of haplotype cluster frequencies in region hapFLK-1c (Chromosome 1, from 18,755,135 to 29,764,967 bp). A: each panel corresponds to a population, from G0 (top) to G5plus (bottom). For one given genomic position (on the x-axis), each color band corresponds to one haplotype cluster, and the height of this band gives the cluster frequency. The selection scenario described in the text is based on cluster frequencies at the position of the strongest hapFLK signal, which is indicated by the vertical black line. B: Evolution of the dark red, red and green cluster frequencies along generations. As discussed in the text, these clusters are the ones showing the strongest evidence of selection at this locus. (PNG 1614 kb)

Additional file 3: **Figure S3.** Evolution of haplotype cluster frequencies in region hapFLK-2c (Chromosome 2, from 24,502,152 to 33,353,778 bp). A: each panel corresponds to a population, from G0 (top) to G5plus (bottom). For one given genomic position (on the x-axis), each color band corresponds to one haplotype cluster, and the height of this band gives the cluster frequency. The selection scenario described in the text is based on cluster frequencies at the position of the strongest hapFLK signal, which is indicated by the vertical black line. B: Evolution of the light blue and light green cluster frequencies along generations. As discussed in the text, these clusters are the ones showing the strongest evidence of selection at this locus. (PNG 1608 kb)

Additional file 4: **Figure S4.** Manhattan plot of the BF factor testing the association between SNP and ultimate pH of the pectoralis major (breast) muscle (PM-pHu). Bayes Factor (BF) comprised between 20 (first horizontal dotted line) and 150 (second horizontal dotted line) indicates strong evidence of QTL and BF higher than 150 indicates very strong evidence of QTL. (PDF 198 kb)

Additional file 5: **Figure S5.** Manhattan plot of the BF factor testing the association between SNP and ultimate pH of the sartorius (thigh) muscle (SART-pHu). Bayes Factor (BF) comprised between 20 (first horizontal dotted line) and 150 (second horizontal dotted line) indicates strong evidence of QTL and BF higher than 150 indicates very strong evidence of QTL. (PDF 198 kb)

Additional file 6: **Table S1.** Comparison of *p*-values (in -log10 scale) for different tests rejecting neutral evolution, for the most significant SNP of the 10 regions listed in Table 2. (DOCX 14 kb)

Additional file 7: **Table S2.** Differential expressed genes within the most significant signatures of selection and QTL regions*. (DOCX 16 kb)
