# Supplementary figures and images for: Identification of genomic regions and candidate genes for chicken meat ultimate pH by combined detection of selection signatures and QTL
Source: BMC Genomics. 2018 Apr 25;19:294. doi: 10.1186/s12864-018-4690-1 (PMC5918591; doi:10.1186/s12864-018-4690-1)

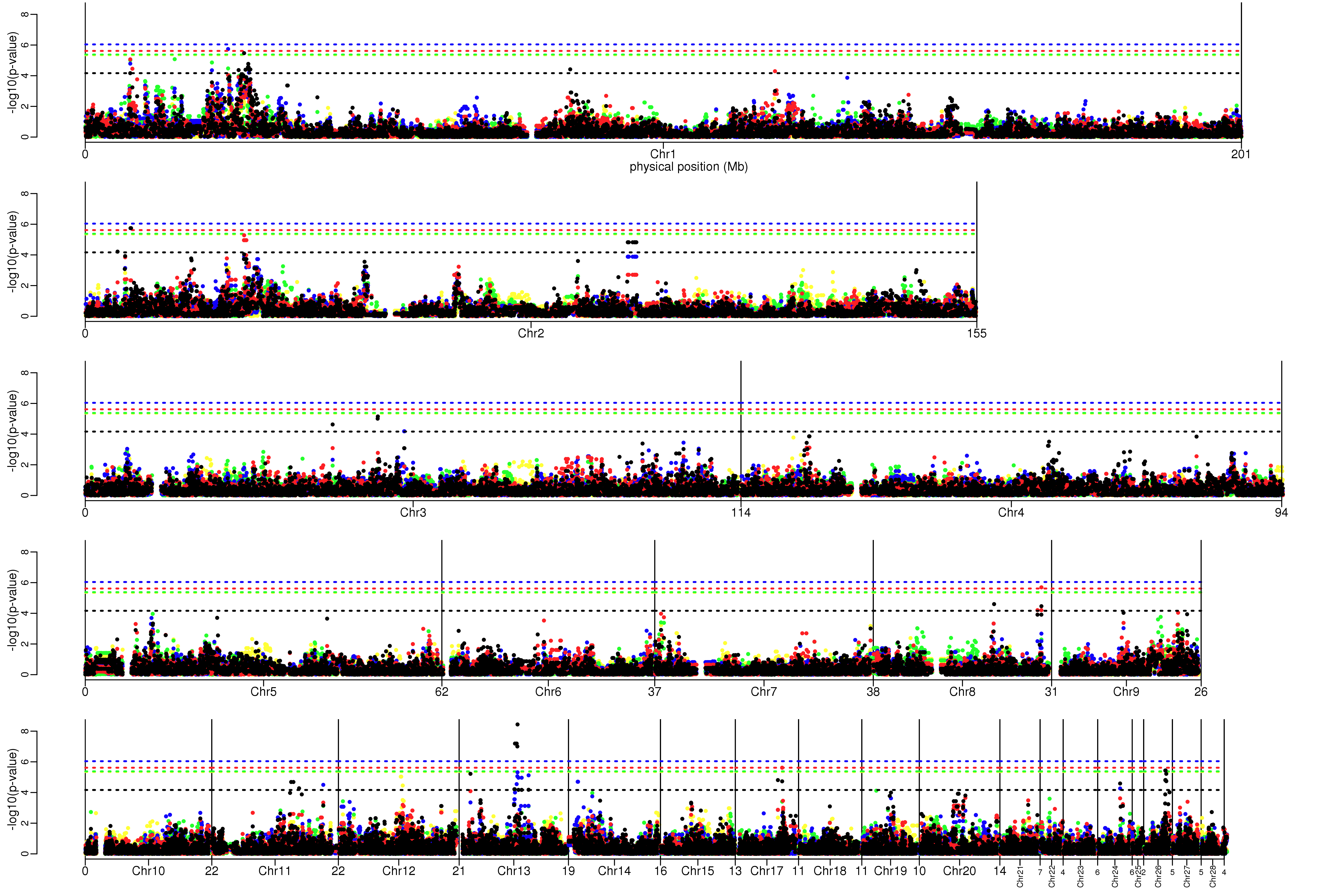

Supplement: Supplementary file 1 — Figure S1. p-values (in -log10 scale) obtained along the genome when applying FLK to generation 1 (yellow), 2 (green), 3 (blue), 4 (red) or 5 (black) of the selection experiment. Horizontal dotted lines indicate the 5% FDR threshold for each of these generations. Vertical black lines correspond to a change of chromosome, and the numbers below these lines provide the length of the finishing chromosome. (TIFF 1599 kb) [file 12864_2018_4690_MOESM1_ESM.tiff]

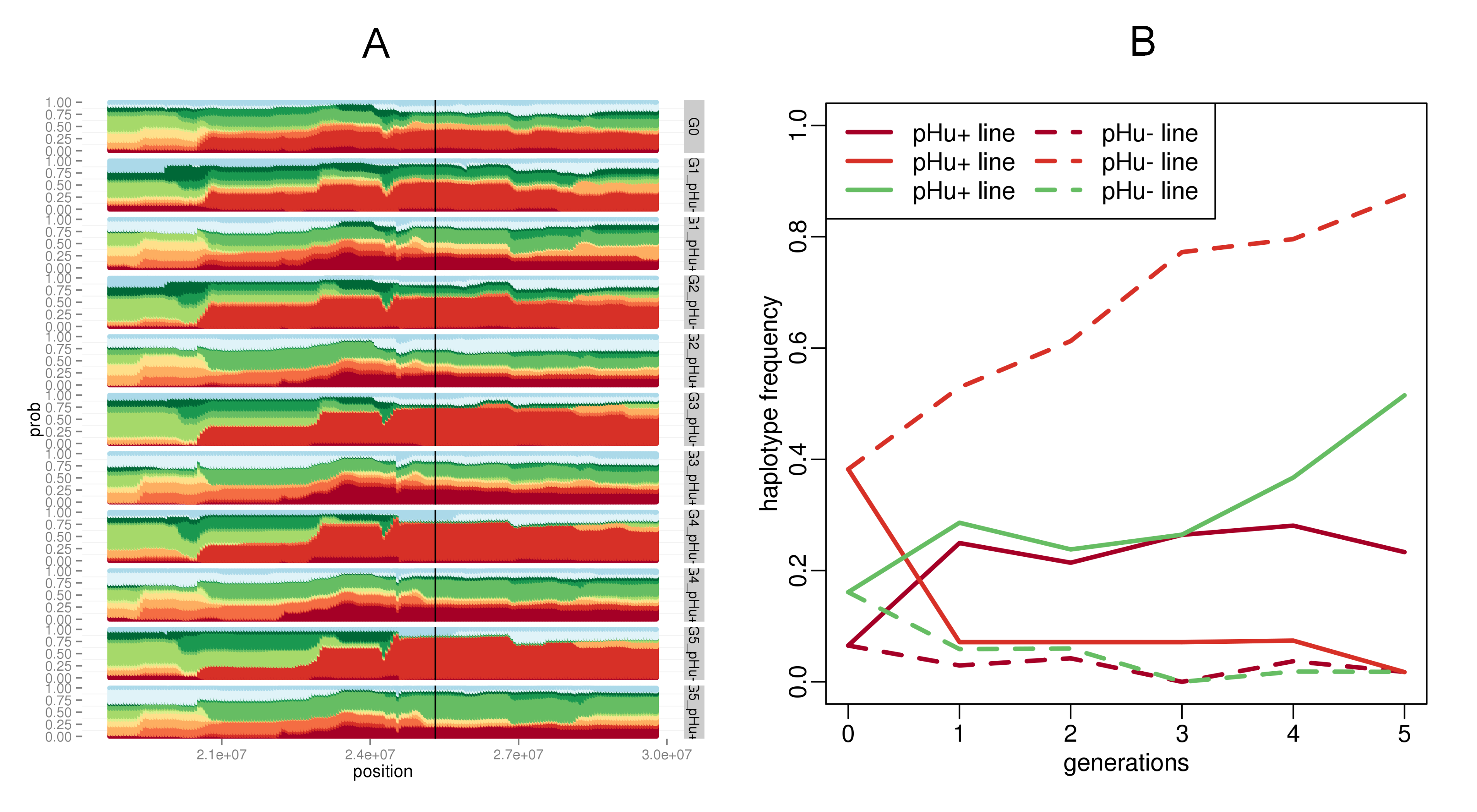

Supplement: Supplementary file 2 — Figure S2. Evolution of haplotype cluster frequencies in region hapFLK-1c (Chromosome 1, from 18,755,135 to 29,764,967 bp). A: each panel corresponds to a population, from G0 (top) to G5plus (bottom). For one given genomic position (on the x-axis), each color band corresponds to one haplotype cluster, and the height of this band gives the cluster frequency. The selection scenario described in the text is based on cluster frequencies at the position of the strongest hapFLK signal, which is indicated by the vertical black line. B: Evolution of the dark red, red and green cluster frequencies along generations. As discussed in the text, these clusters are the ones showing the strongest evidence of selection at this locus. (PNG 1614 kb) [file 12864_2018_4690_MOESM2_ESM.png]

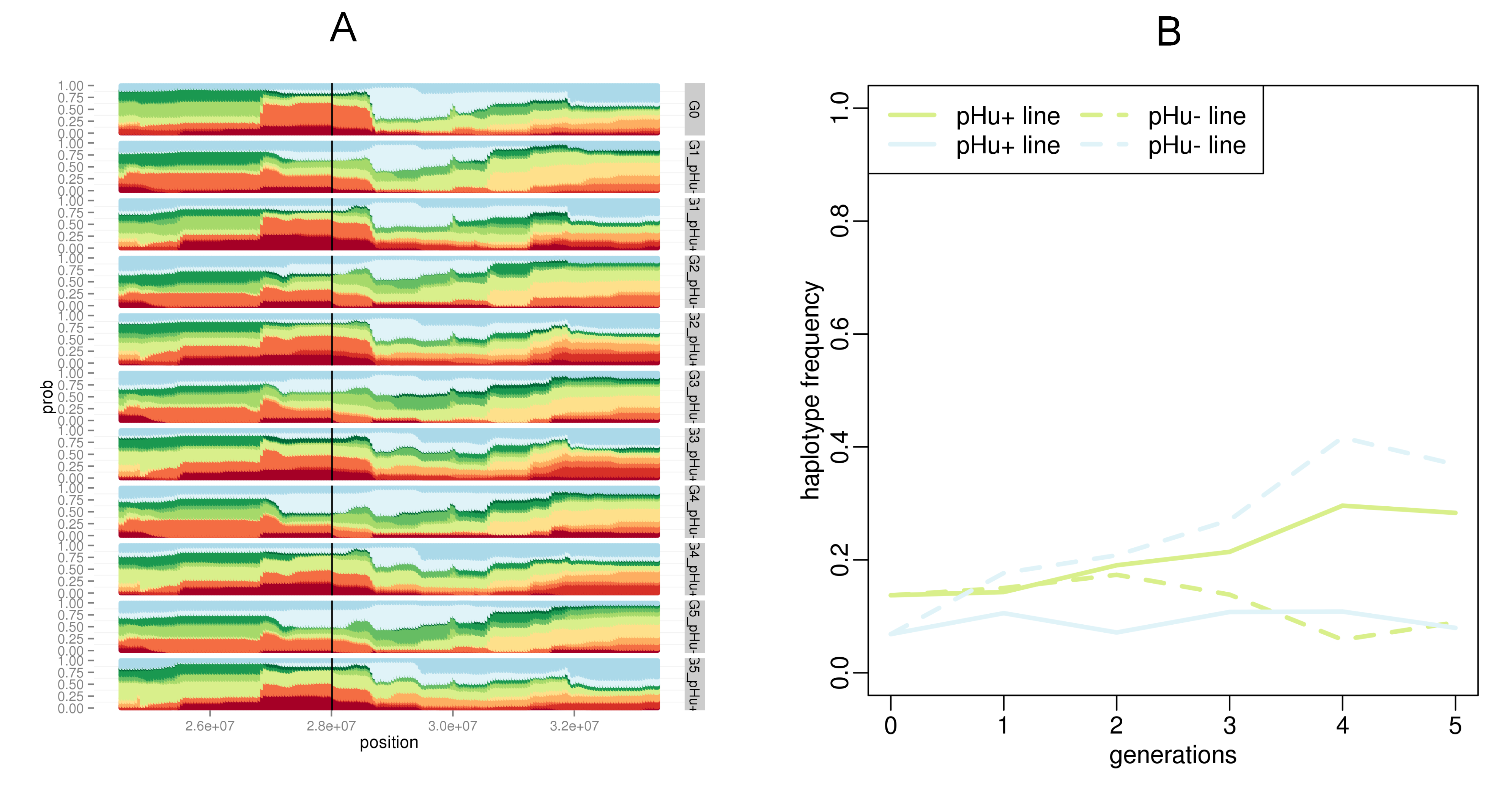

Supplement: Supplementary file 3 — Figure S3. Evolution of haplotype cluster frequencies in region hapFLK-2c (Chromosome 2, from 24,502,152 to 33,353,778 bp). A: each panel corresponds to a population, from G0 (top) to G5plus (bottom). For one given genomic position (on the x-axis), each color band corresponds to one haplotype cluster, and the height of this band gives the cluster frequency. The selection scenario described in the text is based on cluster frequencies at the position of the strongest hapFLK signal, which is indicated by the vertical black line. B: Evolution of the light blue and light green cluster frequencies along generations. As discussed in the text, these clusters are the ones showing the strongest evidence of selection at this locus. (PNG 1608 kb) [file 12864_2018_4690_MOESM3_ESM.png]

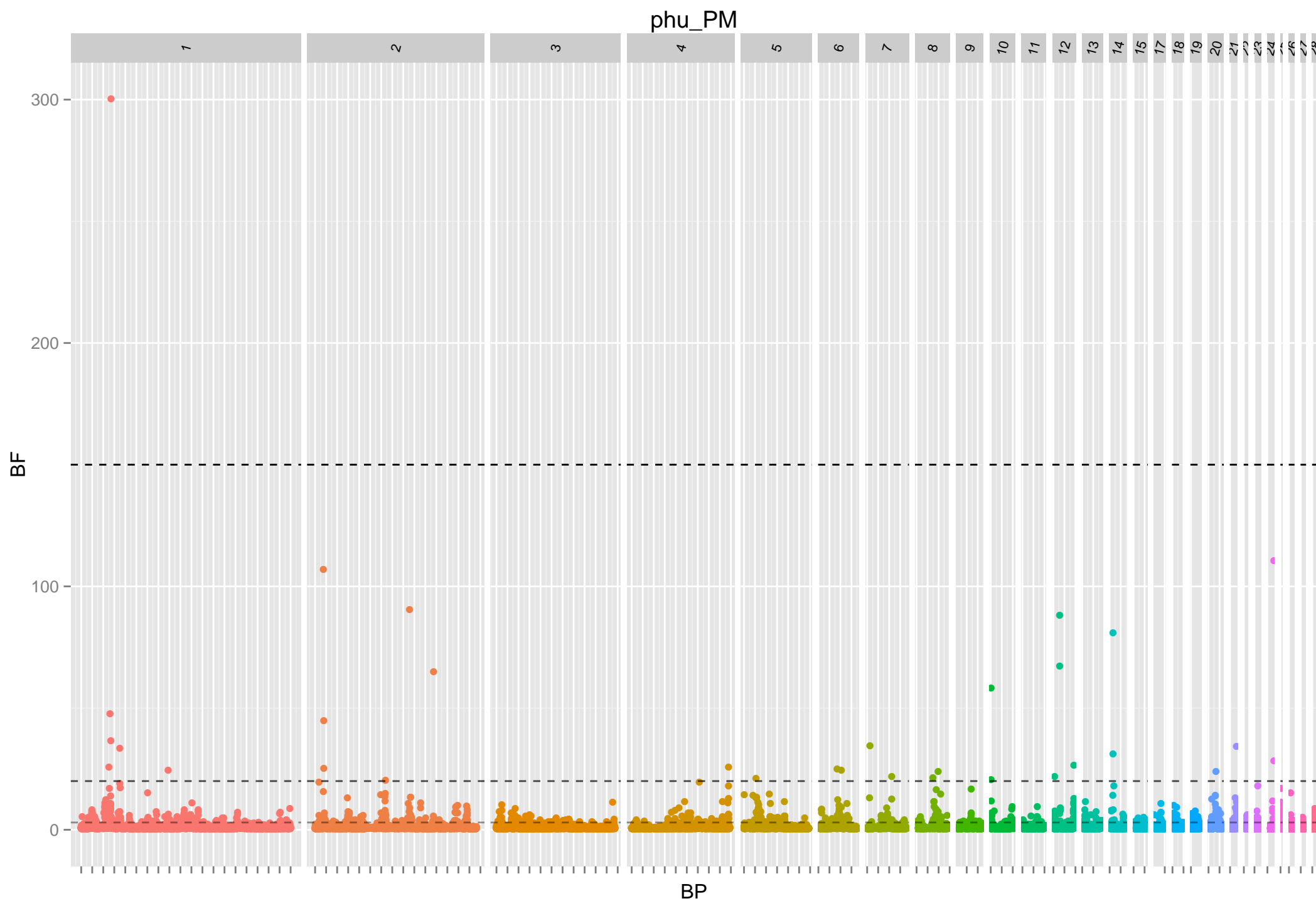

Supplement: Supplementary file 4 — Figure S4. Manhattan plot of the BF factor testing the association between SNP and ultimate pH of the pectoralis major (breast) muscle (PM-pHu). Bayes Factor (BF) comprised between 20 (first horizontal dotted line) and 150 (second horizontal dotted line) indicates strong evidence of QTL and BF higher than 150 indicates very strong evidence of QTL. (PDF 198 kb) [file 12864_2018_4690_MOESM4_ESM.pdf]

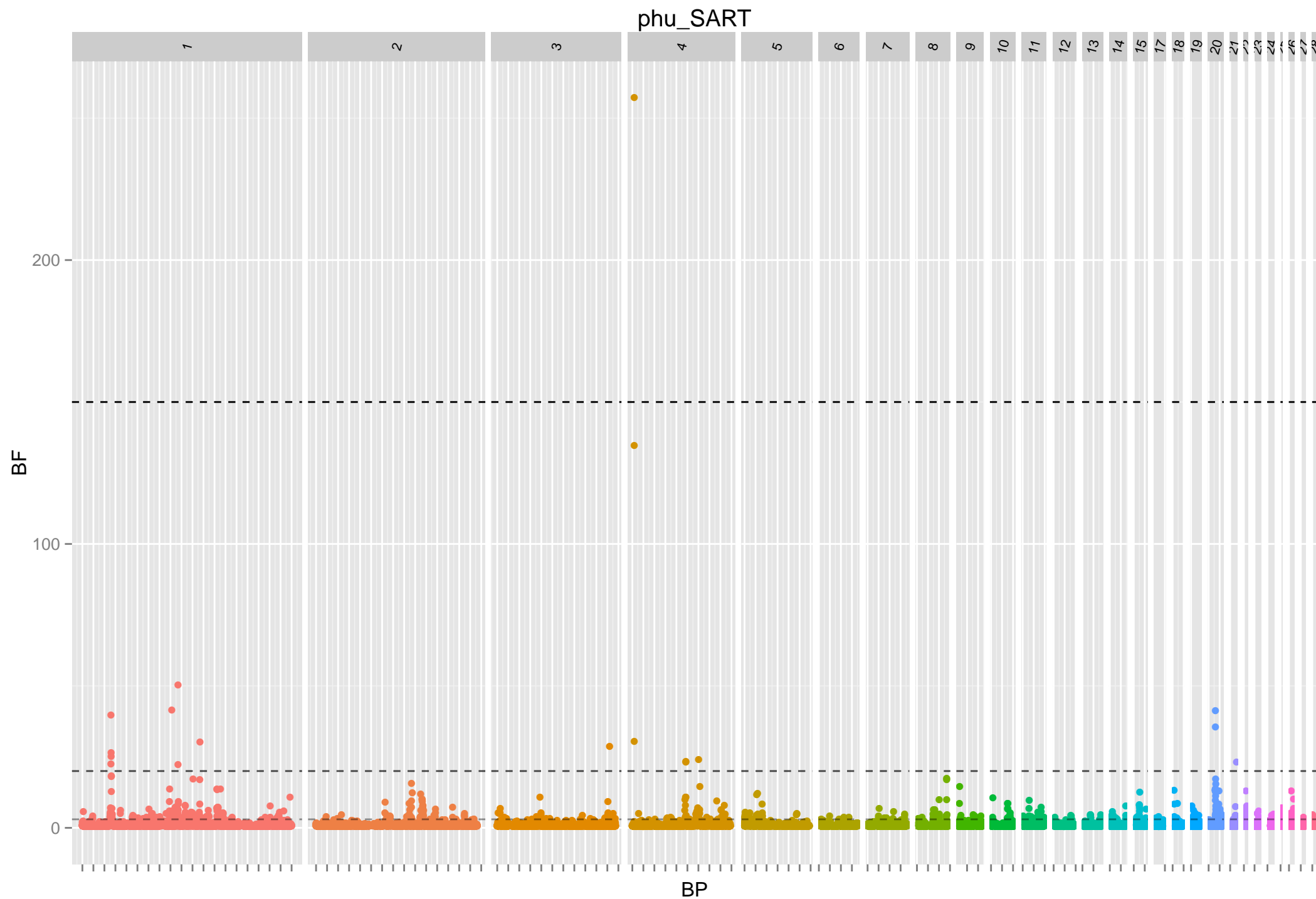

Supplement: Supplementary file 5 — Figure S5. Manhattan plot of the BF factor testing the association between SNP and ultimate pH of the sartorius (thigh) muscle (SART-pHu). Bayes Factor (BF) comprised between 20 (first horizontal dotted line) and 150 (second horizontal dotted line) indicates strong evidence of QTL and BF higher than 150 indicates very strong evidence of QTL. (PDF 198 kb) [file 12864_2018_4690_MOESM5_ESM.pdf]
