# Supplementary material for: Identification of genomic regions and candidate genes for chicken meat ultimate pH by combined detection of selection signatures and QTL
Source: BMC Genomics. 2018 Apr 25;19:294. doi: 10.1186/s12864-018-4690-1 (PMC5918591; doi:10.1186/s12864-018-4690-1)
Supplement: Supplementary file 6 — Table S1. Comparison of p-values (in -log10 scale) for different tests rejecting neutral evolution, for the most significant SNP of the 10 regions listed in Table 2. (DOCX 14 kb) [file 12864_2018_4690_MOESM6_ESM.docx]

**Additional Table 1. Comparison of p-values (in -log10 scale) for different tests rejecting neutral evolution, for the most significant SNP of the 10 regions listed in Table 2.**

| Region name | Allele frequency  in G0 | FLK | Wright-Fisher simulations | Simulations  accounting for the number of reproducers. |
| --- | --- | --- | --- | --- |
| FLK-2a | 0.12 | 4.21 | 4.09 | 7.00 |
| FLK-2b | 0.01 | 4.82 | 2.45 | 3.94 |
| FLK-3a | 0.07 | 4.63 | 3.54 | 5.45 |
| FLK-8a | 0.07 | 4.60 | 3.63 | 5.16 |
| FLK-8b | 0.02 | 4.46 | 2.77 | 4.10 |
| FLK-11a | 0.04 | 4.69 | 4.63 | 6.67 |
| FLK-13a | 0.03 | 5.22 | 3.10 | 5.05 |
| FLK-13b | 0.05 | 8.44 | 4.70 | Inf* |
| FLK-17a | 0.02 | 4.81 | 2.95 | 4.12 |
| FLK-24a | 0.02 | 4.59 | 2.58 | 4.31 |

* p-value is 0
