# Supplementary material for: Identification of genomic regions and candidate genes for chicken meat ultimate pH by combined detection of selection signatures and QTL
Source: BMC Genomics. 2018 Apr 25;19:294. doi: 10.1186/s12864-018-4690-1 (PMC5918591; doi:10.1186/s12864-018-4690-1)
Supplement: Supplementary file 7 — Table S2. Differential expressed genes within the most significant signatures of selection and QTL regions*. (DOCX 16 kb) [file 12864_2018_4690_MOESM7_ESM.docx]

**Additional Table 2. Differential expressed genes within the most significant signatures of selection and QTL regions*.**

| Name of the region | Chromosome (beginning-end of the region) | Number of genes within the region | Number of DE genes# out of the number of genes present on microarray in the region | Names of the DE genes# |
| --- | --- | --- | --- | --- |
| HapFLK-1b | 1 (7050235-16305108) | 103 | 7/57 | DMTF1, MAGI2, ORC5, RINT1, COG5, BCAP29, LRRK2 |
| hapFLK-1c | 1 (18755135-29764967 | 108 | 10/55 | PLXNB2, LMOD2,  ATP6AP1, CTTNBP2,  CAPZA2, PPP1R3A,  LOC107052650,  LSMEM1, IFRD1,  NRCAM |
| hapFLK-1f | 1 (145728258-147459365) | 16 | 1/7 | ABCC4 |
| hapFLK-2a | 2 (7923417-10281724) | 21 | 2/12 | UBE3C, WDR60 |
| hapFLK-2c | 2 (24502152-33353778) | 128 | 4/61 | HOXA9, HOXA10,  HIBADH, TAX1BP1 |
| hapFLK-2e | 2 (118779538-121679302) | 25 | 1/11 | ZC2HC1A |
| hapFLK-5a | 5 (10152843-11215390) | 17 | 1/8 | PDE3B |
| hapFLK-9e | 9 (19461244-21431722) | 46 | 4/21 | PHC3, GPR160,  SERPINI1, PDCD10 |
| hapFLK-26a | 26 (3525075-4343150) | 48 | 5/35 | RHOC, DENND2C,  AMPD1, SIKE1,  TSPAN2 |
| FLK-13b | 13 (9083535-12423603) | 108 | 8/54 | SFXN1, KIF20A,  NHP2, RGS14,  RAB24, SIMC1,  SPINK5, LSM11 |
| QTL SART-4a | 4 (914424-2914424) | 129 | 3/71 | OCRL, MPP1, LRCH2 |
| QTL PM-14a | 14 (2387115-4387115) | 56 | 6/36 | ELFN1, FTSJ2,  NUDT1, BRAT1,  GNA12, MMD2 |
| QTL PM-24a | 24 (5153126-7153126) | 67 | 5/37 | SIDT2, FXYD6,  MIR34B, SLC37A4, NCAM1 |

* The ten most significant signatures of selection, detected by HapFLK or FLK methodology with a p-value below 1e-6, were kept in this top list of the most interesting regions. Out of the 4 most significant SNP (with BF value higher than 100), two were positioned within already kept signatures of selection (Gga_rs13842050 at position 26895425 on GGA1 within hapFLK-1c and Gga_rs14135370 at position 8052991 on GGA2 within hapFLK-2a). The two others were located at position 1914424 on GGA4 and 6153126 on GGA24 which corresponded to QTL regions named SART-4a and PM-24a, respectively. The last QTL region we retained in this top list was the 2Mb region surrounding GGaluGA100179 marker on GGA14 (QTL PM-14a). It was detected with a BF factor of 80 but was positioned less than 1Mb away from hapFLK-14a region. # These numbers and names of genes are issued from the transcriptomic analysis of the pectoralis major muscle which evidenced a list of 1436 differentially expressed (DE) genes between the two lines [23].
